# JNK Inactivation Induces Polyploidy and Drug-Resistance in Coronarin D-Treated Osteosarcoma Cells

**DOI:** 10.3390/molecules23092121

**Published:** 2018-08-23

**Authors:** Chang-Te Hsu, Yi-Fu Huang, Chen-Pu Hsieh, Chia-Chieh Wu, Tai-Shan Shen

**Affiliations:** 1 Department of Orthopedic Surgery, Changhua Christian Hospital, Changhua 50006, Taiwan; 169485@cch.org.tw (C.-T.H.); 51114@cch.org.tw (C.-P.H.); 2 Orthopedics & Sports Medicine Laboratory, Changhua Christian Hospital, Changhua 50006, Taiwan; 181064@cch.org.tw; 3 Institute of Biomedical Sciences, National Chung Hsing University, 145 Xingda Rd., South Dist, Taichung 40227, Taiwan; 4 School of Medicine, Kaohsiung Medical University, Kaohsiung 80708, Taiwan

**Keywords:** coronarin D, JNK, osteosarcoma

## Abstract

Inhibition of proliferating cells is a critical strategy for cancer therapy. In this study, we demonstrated that coronarin D, a natural component extracted from the rhizomes of *Hedychium coronarium*, significantly suppressed the proliferation of osteosarcoma cells. The treatment with coronarin D resulted in the activation of caspase-3 and apoptosis. This treatment induced the accumulation of cyclin B1 and DNA condensation indicating the treated osteosarcoma cells were arrested in mitotic phase. Furthermore, the treatment with coronarin D increased the levels of phosphorylated c-Jun NH2-terminal kinase (JNK) in human osteosarcoma cells. Pretreatment with JNK inhibitor blocked the accumulation of cyclin B1 and DNA condensation, resulting the accumulation of tetraploid cells in coronarin D-treated osteosarcoma HOS cells, indicating JNK inactivation blocked the mitotic entry and arrested cells in the 4 N state. After adaptation, the arrested tetraploid cells continued to duplicate their DNA resulting in polyploidy. Interestingly, when the arrested mitotic cells induced by coronarin D were treated with JNK inhibitor, the accumulated cyclin B1 and DNA condensation were immediately eliminated. These arrested 4 N cells loss the ability to undergo cytokinesis, and ultimately continued to duplicate DNA upon prolonged arrest resulting in the production of polyploid populations. JNK inactivation, either by the pretreatment with JNK inhibitor or the treatment with JNK inhibitor in coronarin D-induced mitotic cells, both caused resistance to coronarin D-induced cell death. Taken together, our findings indicate that coronarin D induces the apoptosis and mitosis arrest in human osteosarcoma cells. JNK has a crucial role in coronarin D-induced mitosis arrest and apoptosis. We hypothesize that functional evaluation of JNK may produce more specific and effective therapies in coronarin D-related trail for treatment of human osteosarcoma.

## 1. Introduction

Osteosarcoma is the most common type of primary malignant bone tumor. It arises from osteoid tissue in the bone during periods of rapid growth and predominately affects adolescents and young adults. Current osteosarcoma treatments include surgical resection, chemotherapy, and radiotherapy. The 5-year survival rate for patients remains at 60–70%. Osteosarcoma is characterized by a high propensity for metastasis. Once this tumor exhibits the ability to invade, the 5-year survival rate for patients with metastatic osteosarcoma decreases dramatically to approximately 20%. It has remained virtually unchanged over the past 30 years [1,2]. Therefore, there is an urgent need for newer effective cures for patients with osteosarcoma, especially for patients suffering from advanced osteosarcoma.

Recent progress has focused on the chemoprevention by natural products for their antigrowth activity against cancer cells [3]. These compounds may exhibit less side effects compared to synthetic chemicals. For example, taxol is isolated from the bark of Taxus brevifolia. It is a microtubule-stabilizing agent used to treat a number of types of cancers including ovarian, breast, and lung cancer [4,5]. Coronarin D, a natural product extracted from the rhizomes of *Hedychium coronarium*, has been shown to possess antimicrobial and antifungal activity [6,7]. Moreover, it has proven to have anti-inflammatory effects and apoptosis potential in cells [8]. The activity of coronarin D in antitumor is unclear. In this study, we evaluated the potential antitumor effect of coronarin D against osteosarcoma.

## 2. Results

### 2.1. Coronarin D Reduced Osteosarcoma Cell Viability and Proliferation

**Figure 1:**
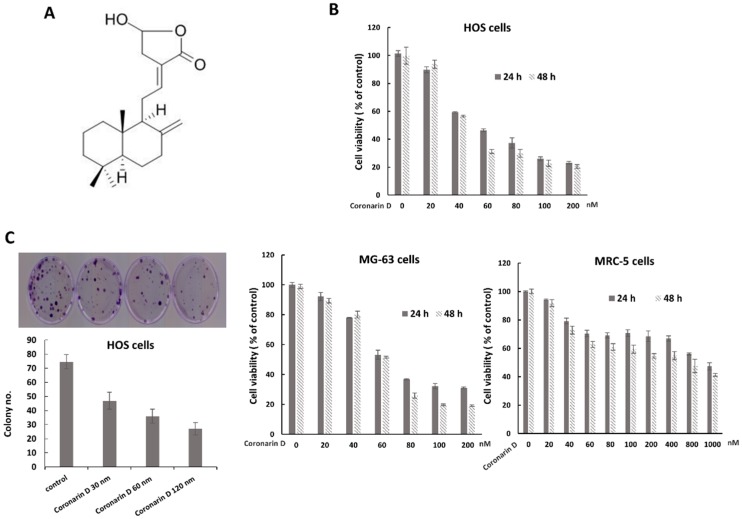
Coronarin D inhibits cell viability of osteosarcoma. (**A**) The chemical structure of coronarin D. (**B**) Coronarin D inhibits osteosarcoma cell growth in a dose- and time-dependent manner. MTT assays were performed with osteosarcoma HOS and MG-63 cells, or human fibroblast cell line MRC-5 exposed to coronarin D in the indicated concentations. Data are expressed as mean ± SD of three independent experiments. (**C**) Coronarin D reduces colony formation of osteosarcoma. HOS cells were plated in colony formation assays after treatment with coronarin D for 6 h. Five-hundred cells were plated per dish. All experiments were performed in triplicate, and the figure above shows a representative example.

Coronarin D is a labdane-type diterpene (Figure 1A). Cell Viability of osteosarcoma cells after exposure to various concentrations of coronarin D (0–200 nM) was tested by MTT assay. The results showed that coronarin D significantly inhibited the growth of osteosarcoma HOS cells and MG-63 cells in a dose- and time-dependent manner, and had a minor cytotoxic effect in human fibroblast cell line MRC-5. (Figure 1B). The half maximal inhibitory concentration (IC_50_) calculated based on data of the MTT assays for HOS cells were 58.8 nM at 24 h and 51.18 nM at 48 h, those for MG-63 cells were 65.87 nM at 24 h and 61.9 nM at 48 h, and those for MRC-5 cells were 697.39 nM at 24 h and 492.42 nM at 48h. We also examined the effect of coronarin D on cell proliferative capacity by colony formation assays. The results showed that the treatment of coronarin D reduced colony number in a dose-dependent manner in osteosarcoma HOS cells. These data indicate that coronarin D has the potential to reduce the viability of osteosarcoma cells.

### 2.2. Coronarin D Induces Apoptosis and Cell Arrest

**Figure 2:**
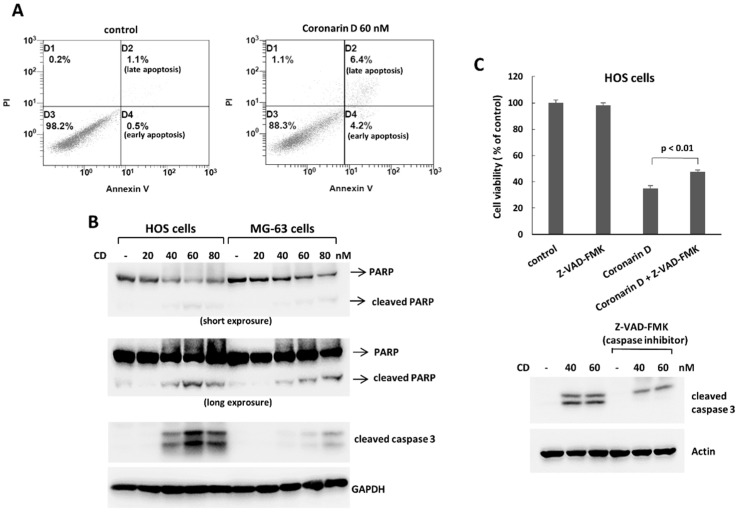
Coronarin D induces apoptosis in osteosarcoma cells. Osteosarcoma cells were treated with coronarin D for 24 h. To detect apoptosis, the HOS cells were stained with Annexin V and propidium iodide, and analyzed using flow cytometry (**A**). Expression of apoptosis-related proteins was measured by Western blotting in HOS cells and MG-63 cells (**B**). (**C**) Caspase inhibitor blocks the antiproliferative effect caused by coronarin D treatment. HOS cells were treated with coronarin D (60 nM) for 24 h in the absence or presence of caspase inhibitor, ZVAD-FMK (10 μM). The treated cells were processed with Western blotting to analyze the caspase 3 activation and MTT assay to determine cell viability.

To determine whether programmed cell death was involved in the antiproliferative effect of coronarin D, we analyzed cell for apoptosis by Annexin V staining and the expression of apoptosis-related proteins by Western blotting. Coronarin D treatment induced a significant increase of Annexin V positive cells (Figure 2A), and upregulated the expressions of apoptosis-related proteins, cleaved caspase 3, and cleaved PARP in human osteosarcoma cells (Figure 2B). The data indicate that coronarin D treatment induces apoptosis in human osteosarcoma cells. Furthermore, the caspase inhibitor, ZVAD-FMK, rescued the decreased cell survival caused by coronarin D treatment (Figure 2C). These data indicate that coronarin D suppresses cell survival by caspase-dependent apoptosis.

**Figure 3:**
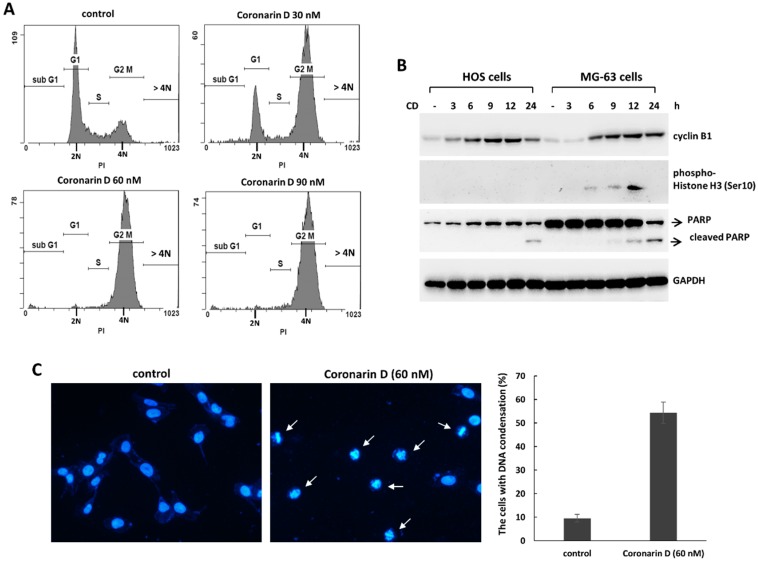
Coronarin D induces M phase arrest in osteosarcoma cells. HOS cells were treated with indicated concentrations of coronarin D for 12 h. The cells were stained with propidium iodide and analyzed by flow cytometer (**A**). (**B**) Coronarin D induces the accumulation of mitosis phase marker in osteosarcoma cells. HOS cells and MG-63 cells were treated with coronarin D (60 nM). The treated cells were harvested in the indicated time points, and analyzed by Western blotting. (**C**) Coronarin D induced DNA condensation in HOS cells. HOS cells were treated with coronarin D (60 nM) for 6 h. The cells were stained with 4,6-diamidino-2-phenylindole (DAPI) for labeling nucleus (blue color), and mitosis cells (white arrows) were observed under microscopic analysis.

The analysis of cell cycle distribution in treated cells were performed by PI staining and flow cytometry. The data showed the number of 4 N cells were significantly increased in coronarin D-treated HOS cells indicating coronarin D may arrest cells in mitosis (Figure 3A). To confirm the state of 4 N cells accumulated by the treatment of coronarin D, the mitosis markers, cyclin B1 and phospho-histone H3 at Ser10, were analyzed in coronarin D-treated osteosarcoma cells. The data showed that cyclin B1 was significantly increased in HOS and MG-63 cells, and phospho-histone H3 at Ser10 was increased in MG-63 cells after the exposure of coronarin D (Figure 3B). The number of cells with DNA condensation were increased in coronarin D-treated HOS cells (Figure 3C). Therefore, the 4 N cells accumulated by the treatment of coronarin D should be arrested in mitotic phase. Taken together, these data indicate that coronarin D suppresses cell proliferation by apoptosis and mitosis arrest in human osteosarcoma cells.

### 2.3. The Pretreatment with JNK Inhibitor Blocks the Mitotic Entry and Induces Polyploidy and Drug-Resistance in Coronarin D-Treated HOS Cells

**Figure 4:**
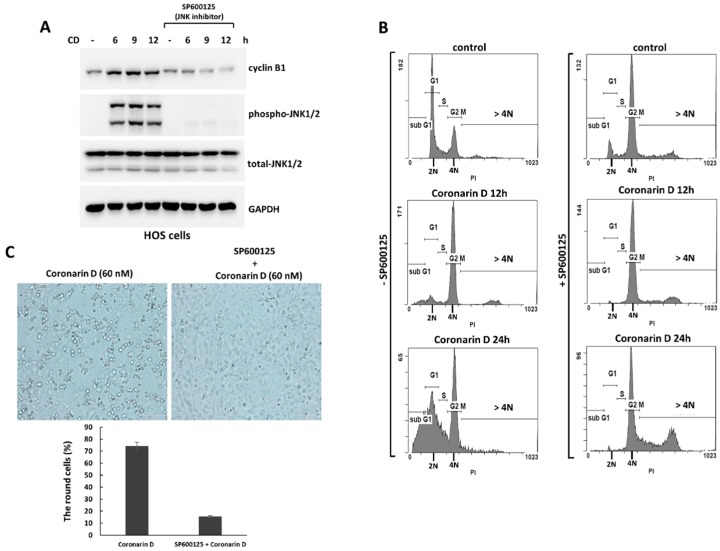
JNK inactivation blocks mitotic entry and induces polyploidy in coronarin D-treated HOS cells. (**A**) JNK inhibitor inhibited the accumulation of cyclin B1 in HOS cells. HOS cells were pretreated with JNK inhibitor, SP600125 (20 μM) for 1 h, and cotreated with coronarin D (60 nM) and harvested in the indicated time points. The treated cells were analyzed by Western blotting using the indicated antibodies. (**B**) JNK inhibitor induces polyploidy in coronarin D-treated HOS cells. HOS cells were treated as described in (**A**). The treated cells were harvested in the indicated time points. The cells were stained with propidium iodide and analyzed by a flow cytometer. (**C**) Round mitotic cells were greatly reduced by cotreatment with SP600125. Phase-contrast photomicrographs of HOS cells treated with coronarin D (60 nM) in the absence or presence of JNK inhibitor (SP600125) (20 μM) for 12 h.

**Figure 5:**
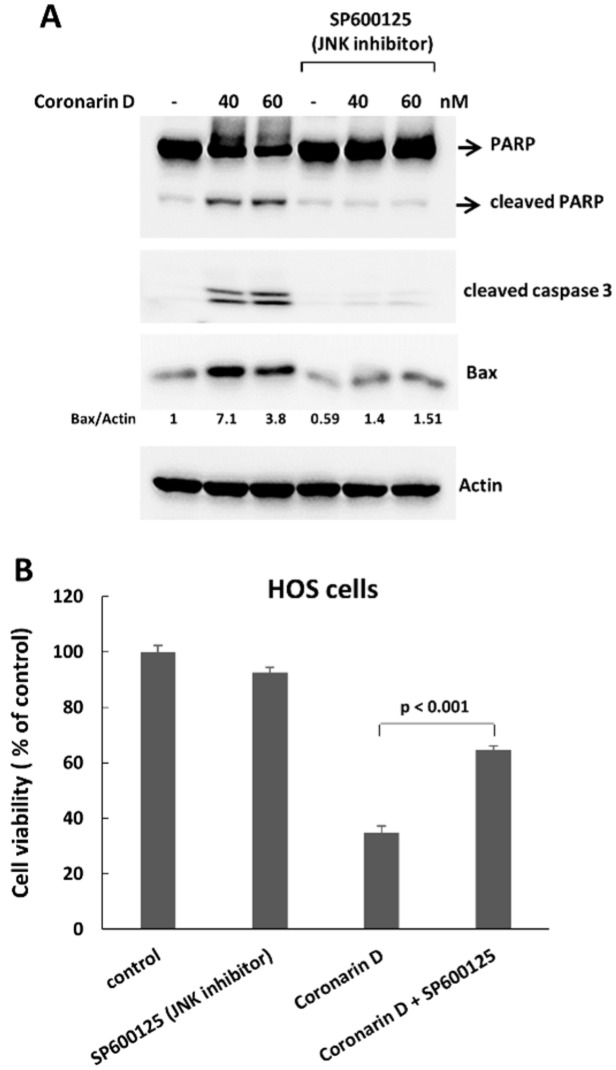
JNK inactivation inhibits coronarin D-induced cell death. (**A**) JNK inactivation suppresses coronarin D-induced cell death. HOS cells were pretreated with JNK inhibitor (SP600125) (20 μM) for 1 h, and cotreated with coronarin D for 24 h. The treated cells were processed with Western blotting to analyze the caspase 3 activation and Bax level. Numbers indicate relative levels of Bax after normalization to Actin. (**B**) JNK inhibitor blocks the antiproliferative effect caused by coronarin D treatment. HOS cells were pretreated with JNK inhibitor (SP600125) (20 μM) for 1 h, and cotreated with coronarin D (80 nM) for 24 h. The treated cells were processed with MTT assay to determine cell viability.

MAPK signaling plays an important role in many cellular processes including cell division, differentiation, proliferation, and apoptosis [9]. In this study, we found that treatment of coronarin D resulted in increased expression of phospho-JNK following the accumulation of cyclin B1 (Figure 4A). To demonstrate that the role of JNK activation in coronarin D-triggered mitosis arrest and apoptosis, we pretreated HOS cells with the JNK inhibitor (SP600125) (20 μM) for 1 h, and then cotreated cells with coronarin D. This inhibitor induced the accumulation of 4 N cells (Figure 4B) without increased cyclin B1 (Figure 4A) and rounded mitotic cells (Figure 4C) suggesting that JNK inactivation blocked the mitotic entry of coronarin D-treated HOS cells and arrested the cells in 4 N state. After adaption, the arrested 4 N cells continued to duplicate their DNA resulting in the production of polyploid population (Figure 4B). Importantly, JNK inactivation inhibited the formation of cleaved caspase3, PARP, and JNK downstream targets, Bax (Figure 5A) in the coronarin D-treated HOS cells, and blocked the antiproliferative effect induced by coronarin D (Figure 5B). We therefore conclude JNK inactivation cause the resistance to coronarin D-induced cell death. 

### 2.4. JNK Inactivation Eliminates the Accumulated Cyclin B1 and Induces Polyploidy in Coronarin D-Induced M Phase Arrest Cells

**Figure 6:**
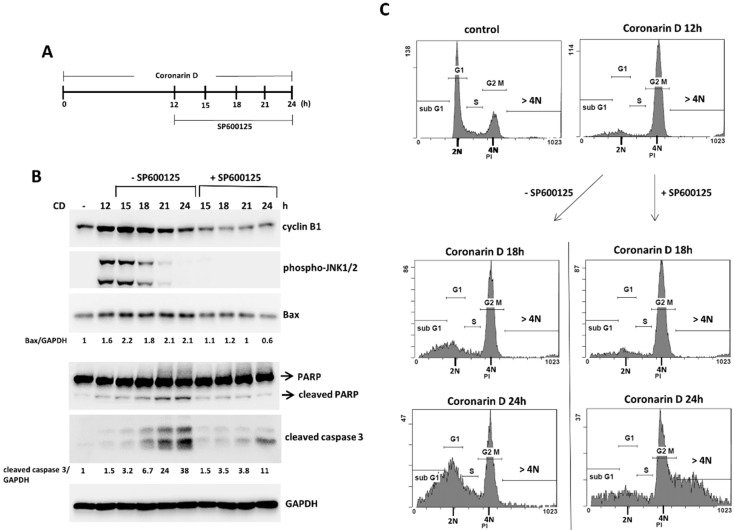
JNK inactivation eliminates the accumulation of cyclin B1 and induces polyploid in coronarin D-induced M phase arrest cells. (**A**) Schedule of drug treatment. HOS cells were treated with coronarin D for 12 h, and coronarin D treatment was continued with or without JNK inhibitor (SP600125) (20 μM). The treated cells were harvested at the indicated time points and analyzed by Western blotting using the indicated antibodies (**B**) and by flow cytometry (**C**). Numbers indicate relative levels of Bax or cleaved caspase 3 after normalization to GAPDH (**B**).

To precisely analysis the role of JNK activation in coronarin D-induced mitosis, We first treated cells with coronarin D for 12 h to induce the accumulation of mitotic cells, and then cotreated with JNK-specific inhibitor (SP600125) (20 μM) in these arrested mitotic cells (Figure 6A). Once JNK activity was blocked in the arrested mitotic cells, the accumulated cyclin B1 (Figure 6B) and DNA condensation (the data not shown) were immediately eliminated. These arrested 4 N cells lost the ability to undergo cytokinesis, and ultimately continued to duplicate DNA upon prolonged arrest resulting in the production of polyploid populations and drug resistance toward lower level of activated caspase 3 (Figure 6B,C). These data support the hypothesis the JNK activation play an essential role in the coronarin D-induced mitotic arrest and apoptosis.

## 3. Discussion

Coronarin D, a natural product extracted from the rhizomes of *Hedychium coronarium*, has been shown to possess antimicrobial and antifungal activity [6,7]. It has been proven to have anti-inflammatory effects and apoptosis potential in cells [8]. The antitumor activity of coronarin D is uncertain. Limited work has been published showing that coronarin D induces reactive oxygen species-mediated cell death in human nasopharyngeal cancer cells [10]. In this report, we demonstrated that coronarin D suppressed the proliferation of osteosarcoma cells significantly by mitotic arrest and apoptosis. In addition, we showed that the function of JNK in coronarin D-induced effects.

The JNK pathway is predominantly activated by stress stimuli such as cytokines, growth factors, and ultraviolet irradiation. Although activation of JNK is classically thought to induce cell death, several reports have presented that the activity of JNK is also involved in control of the cell cycle [11,12,13,14]. In breast cancer cells, inhibition of JNK reduces G2/M transit and causes endoreduplication (cellular DNA content >4 N) [15]. In retinal progenitor cells, JNK is phosphorylated preferentially during the early stages of mitosis, and inhibition of JNK induces mitotic arrest [16]. In this report, we showed that the treatment with coronarin D resulted in the accumulation of cyclin B1, DNA condensation, and arrested cells in mitotic phase following JNK activation in osteosarcoma cells. Blocking JNK activation with JNK inhibitor prevented the accumulation of cyclin B1 and rounded morphology, inhibited mitotic progression, and arrested cells in the 4 N state in coronarin D-treated osteosarcoma cells. Upon prolonged arrest, these arrested cells ultimately continued to duplicate DNA resulting in the production of polyploid populations and drug resistance.

It is unclear how JNK regulates mitosis progression. In NIH-3T3 cells, the inactivation of JNK by SP600125 inhibits expression of Aurora B, blocks phosphorylation of Histone H3 at serine 10, and prevents mitosis entry with sustained expression of cyclin B1 [17]. In this report, we observed that the pretreatment with JNK inhibitor prevented the accumulation of cyclin B1 in coronarin D-exposed osteosarcoma cells. Furthermore, in arrested mitotic cells induced by coronarin D, treatment with JNK inhibitor immediately eliminated the accumulated cyclin B1. These results indicated that the roles of JNK activation in mitotic progression may be associated with cyclin B1 in coronarin D-treated osteosarcoma cells.

Our data showed that the treatment with JNK inhibitor suppressed the activation of caspase 3 and rescued the cell viability in coronarin D-treated osteosarcoma cells indicating JNK has crucial role in coronarin D-induced cell death. How did the inactivation of JNK suppress coronarin D-induced cell death? One possibility is that the inhibition of JNK suppresses mitotic entry, thereby preventing coronarin D-induced cell death in mitosis. The formation of polyploidy has been shown to drive resistance to chemotherapy in tumors [18,19,20]. We believe that the polyploidy induced by JNK inactivation may cause resistance to coronarin D-induced cell death in osteosarcoma cells. Taken together with the above data, our findings show that coronarin D induces apoptosis and mitosis arrest in human osteosarcoma cells. JNK has a crucial role in coronarin D-induced mitosis arrest and apoptosis. Functional evaluation of JNK may produce more precise and effective therapies in coronarin D-related trail for treatment of human osteosarcoma.

## 4. Materials and Methods

### 4.1. Cell Culture and Reagents

Human HOS and MG-63 osteosarcoma cells, and human fibroblast cell line MRC-5 were purchased from the Bioresource Collection and Research Center (Hsinchu, Taiwan). MRC-5, HOS, and MG-63 cells were maintained in Minimum Essential Medium (#11095-080; Gibco, Carlsbad, CA, USA) supplemented with 10% fetal bovine serum (Gibco), 1% penicillin/streptomycin (Gibco). JNK inhibitor (SP600125) and z-VAD-FMK were purchased from Santa Cruz Biotechnology. Coronarin D (purity > 95%) was purchased from ChemFaces (Wuhan, Hubei, China).

### 4.2. MTT Assay

The human osteosarcoma HOS and MG-63 cells were seeded in 24-well plates for 24 h. The cells were exposed to different concentrations of carnosol for 24 h. At the end of the assay time, 20 µL of MTT solution (5 mg/mL) (Invitrogen, Carlsbad, CA, USA) was added to each well, and then incubated for 2 h at 37 °C. After removing the cultured medium, 200 µL of dimethyl sulfoxide (DMSO) was added to each well. Absorbance at 590 nm of the dissolved formazan product was read using a spectrophotometric plate reader (Thermo Multiskan SPECTRUM, Thermo Fisher Scientific, Waltham, MA, USA). The half maximal inhibitory concentration (IC_50_) for HOS and MG-63 cells were calculated by the “Forecast” function in Microsoft Excel.

### 4.3. Flow Cytometry Analysis

Cells were fixed with ice-cold 100% ethanol and kept on −20 °C for 24 h. Cells were rehydrated with cold PBS, and then resuspended in PBS with propium iodine (40 µg/mL) (#P4170; Sigma-Aldrich, St. Louis, MO, USA) and Ribonuclease A (0.2 µg/mL) at room temperature for 30 min in the dark. Samples were analyzed by a Cytomics^TM^ FC500 flow cytometer (Beckman Coulter; Brea, CA, USA).

### 4.4. Apoptosis Assay

Cell apoptosis was determined by flow cytometry using the Annexin-V-FITC staining kit (Becton Dickinson, San Jose, CA, USA) according to the manufacturer’s instructions. Briefly, the treated cells were trypsinized, and washed twice by cold PBS. The cells were incubated with 100 μL of 1× binding buffer with 5 μL of FITC Annexin V and 5 μL of propidium iodide for 15 min at room temperature (RT) in the dark. After incubation, 400 μL of 1× binding buffer was added to each tube, and the fluorescence was detected by a Cytomics™ FC500 flow cytometer (Beckman Coulter, Miami, FL, USA).

### 4.5. Cell Lysis and Immunoblotting

Cells were lysed in TEGN buffer (10 mM Tris, pH 7.5, 1 mM EDTA, 420 mM NaCl, 10% glycerol, and 0.5% Nonidet P-40) containing a protease inhibitor cocktail (Roche, Mannheim, Germany), phosphatase inhibitors (Roche), and 1 mM dithiothreitol (DTT). For Western blotting, the cell lysates were boiled in protein sample buffer (2 M β-mercaptoethanol, 12% sodium dodecyl sulfate (SDS), 0.5 M Tris, pH 6.8, 0.5 mg/mL bromophenol blue, and 30% glycerol). The samples were analyzed by SDS-polyacrylamide gel electrophoresis (PAGE). Antibodies used were the following: cleaved caspase-3 (#9661; Cell Signaling, Danvers, MA, USA), actin (A2066; Sigma-Aldrich, St. Louis, MO, USA), PARP (#9542; Cell Signaling), GAPDH (#2118; Cell Signaling), Cyclin B1 (#05-373SP; EMD Millipore, Temecula, CA, USA), SAPK/JNK (#9258; Cell Signaling), phospho-JNK (Thr183/Tyr185, Thr221/Tyr223) (#07-175; EMD Millipore, Temecula, CA, USA), and phospho-histone H3 (Ser10) (#9701; Cell Signaling).

### 4.6. Statistical Analysis

All data were obtained from at least three separate experiments and are expressed as mean ± SD. Statistical comparisons of differences between groups were conducted using the Student’s *t*-test. A *p* value less than 0.05 was considered to represent statistical significance. All statistical analyses were performed using the software package GraphPad Prism (Version 4.0, GraphPad Software; San Diego, CA, USA).
